# Perceptual similarity and clustering in braille letter recognition

**DOI:** 10.1186/s41235-025-00690-x

**Published:** 2026-02-07

**Authors:** Zeynep G. Özkan, Ana Baciero, Manuel Perea, Pablo Gómez

**Affiliations:** 1 Department of Methodology and ERI-Lectura, Universitat de València, Valencia, Spain; 2 Department of Experimental Psychology, Cognitive Processes and Speech Therapy, and Pluridisciplinary Institute, Universidad Complutense de Madrid, Madrid, Spain; 3 Centre of Research Nebrija in Cognition, Universidad Nebrija, Madrid, Spain; 4 Department of Psychology, Skidmore College, 815 N Broadway, Saratoga Springs, NY USA

## Abstract

Braille is a tactile writing system that enables individuals to read through the sense of touch. Although letter recognition research in the visual modality has informed reading instruction debates, the processes underlying braille letter recognition have received comparatively less attention which has led to little input from researchers toward educators. In this study, we first quantified the formal properties of braille dots using measures of cue validity and entropy-based informativeness, and we tested whether the 26 letters of the braille alphabet were linearly separable in the six-dimensional binary space defined by dot presence. We then examined letter discriminability in fluent Spanish braille readers using a same–different task that included all possible letter combinations. From participants’ accuracy and response time data, we constructed perceptual similarity matrices and applied hierarchical clustering to characterize the structure of braille letter similarity. The resulting clusters revealed a structured perceptual space that reflected both local dot features and global configurations. These results provide a characterization of the perceptual structure of the braille alphabet and show constraints on tactile letter recognition that extend beyond dot overlap, offering a benchmark to guide experimental control, instructional sequencing of letters, and computational models of tactile letter recognition.

## Introduction

Some of the most significant contributions of cognitive science research relate to changes, improvement, and examination into educational practices. While reading research has certainly played a role in the literacy and education debates (see Castles et al., 2018; Rayner et al., 2001), there is an entire category of readers that has been understudied: braille readers. This article aims to change that by presenting tools to facilitate research on braille reading and literacy training.

Through raised-dot patterns read with the fingertips, braille allows blind and visually impaired individuals direct access to written language. Like any alphabetic writing system, braille requires readers to identify individual letters to access word meanings.^1^ Letter recognition in the visual modality has been extensively studied (see Carreiras et al., 2014; Dehaene & Cohen, 2011; Grainger et al., 2008; Pelli et al., 2006; Wiley et al., 2016; Testolin et al., 2017). However, the perceptual and cognitive mechanisms underlying tactile letter recognition remain comparatively underexplored. Foundational work by Bertelson and colleagues demonstrated that braille reading can be studied experimentally, establishing the field of tactile reading research (e.g., Mousty & Bertelson, 1985 for work at the sentence level; Bertelson et al., 1992, for work at the word level; and de Heering & Kolinsky, 2019, for work at the letter level). More recently, Baciero et al., (2022, 2023a) and Englebretson et al., (2023, 2024) have renewed interest in braille reading, but experimental research on braille remains the exception rather than the norm. To address this gap, we first examine the formal properties of braille letters and then analyze perceptual data to characterize the structure of tactile letter similarity.

A comprehensive understanding of letter recognition in braille is essential not only for advancing theoretical models of braille reading but more importantly for practical concerns: Braille literacy rates have declined globally (National Federation for the Blind, 2009), with significant implications for quality of life; for instance, employment rates are markedly higher among braille-literate individuals (see Ryles, 1996). Critically, literacy, whether visual or tactile, relies on the fundamental process of “decoding symbols to derive meaning” (Anderson et al., 1985; National Reading Panel, 2000). Thus, examining how letter processing unfolds in the tactile modality offers the opportunity to test the generalizability of cognitive models of reading across sensory channels, while also informing interventions and technologies that support braille literacy in real-world contexts.

### Why braille letter recognition deserves attention

Braille presents unique perceptual constraints and opportunities. Unlike visual letters, braille characters are composed of raised-dot patterns and read sequentially by sliding the fingertips across lines of text.^2^ This requires integrating spatial and temporal information through touch, a very different process from the parallel, high-resolution input typical of visual reading.

Importantly, neuroimaging evidence has revealed that braille and print reading engage overlapping cortical networks involved in orthographic and word processing (see Reich et al., 2011). This convergence suggests a shared cognitive architecture across both sensory modalities, despite the differences in input format. Nonetheless, braille stands apart from other writing systems in significant ways. It was specifically designed for tactile reading, using a 3 × 2 matrix of raised dots that constrains the form of every character. As Millar (2003, p 32) indicates, “Braille characters are bound to be similar to each other since they all derive from the same (3 × 2) matrix.” Furthermore, the physical form of braille letters is highly standardized across contexts: Unlike visual scripts with variable fonts or handwriting styles, braille letters maintain fixed shapes, sizes, and spacing—regulated by international guidelines (ISO, 2013). These parameters define the exact dimensions of dot diameter, spacing, and height to ensure uniformity and readability. Notably, this uniformity may make braille letters particularly susceptible to confusability effects, as even small changes in dot configuration can lead to perceptual overlap. Supporting this idea, Baciero et al. (2023a) showed that pseudowords formed by substituting a single letter with a tactilely similar one (e.g., replacing *g* with f ) produced were more confusable with their base words than those with dissimilar substitutions (e.g., *g* with *s*)—note that a parallel effect occurs in the visual modality with misspelling brand names (i.e., items with a highly consistent format) but not with misspelling common words (see Perea et al., 2022).

**Fig. 1:**
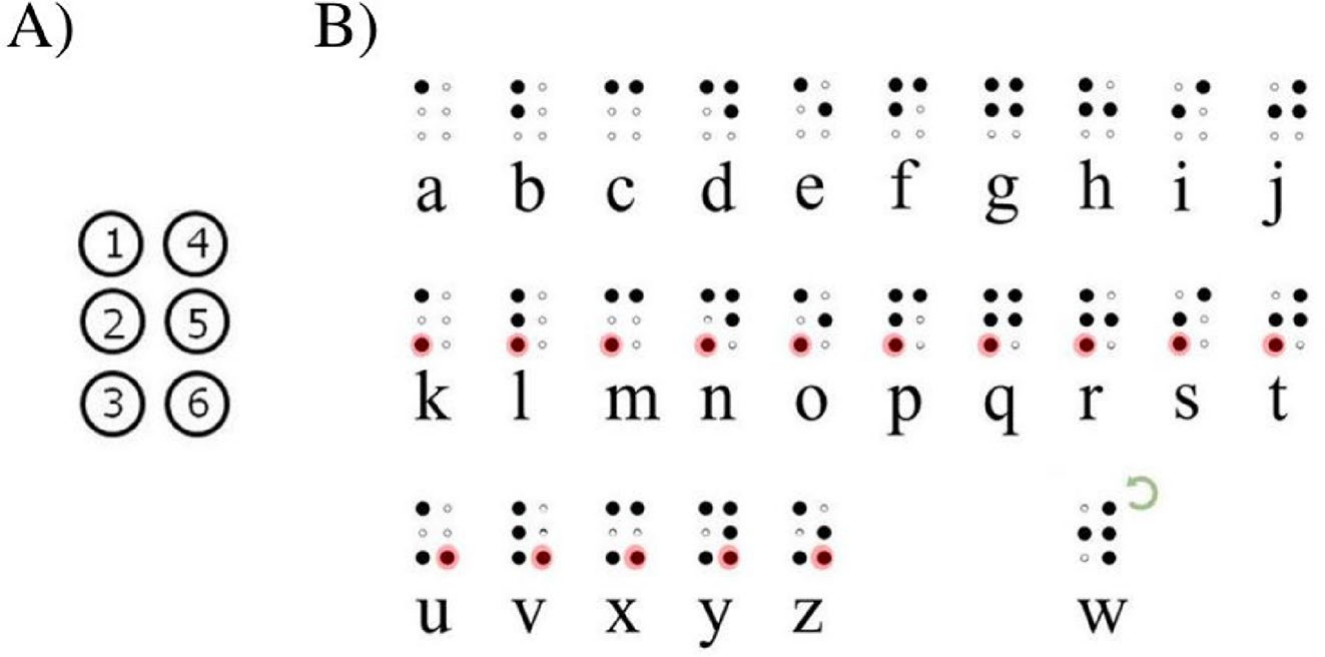
**A** Braille cell and dots’ nomenclature. **B** Braille alphabet as developed by Louis Braille (1829): The first row has the base patterns; the second and third rows build on these base patterns adding dots 3 and 6, respectively (highlighted in red in the graph), which is indicated by the red highlights. The letter *w * does not follow this pattern because, since it did not exist in French, it was added later. It is a rotation of the letter *r *

Louis Braille developed the system in 1829, and it has remained largely unchanged despite the existence of alternative tactile scripts (e.g., Moon System, Moon, 1910; ELIA Frames reading system, Elia, n.d.). Figure 1 illustrates the structure of braille and can be used to explain how Louis Braille devised the system. As can be seen on the top of Fig. 1, the first 10 characters in the alphabet (also known as first decade) are written using the top two rows of dots (*a*, *b*, *c*, *d*, *e*, *f*, *g*, *h*, *i*, *j*), the next ten letters repeat the patterns of the previous ten, adding a dot in the third position (*k*, *l*, *m*, *n*, *o*, *p*, *q*, *r*, *s*, *t*), the next group of letters (*u–z*) also repeat the pattern but add a dot in the sixth position (*u*, v , *x*, *y*, *z*). The letter *w*, not being part of the French alphabet when Louis Braille created this writing system, was later assigned its braille character which is a rotation of *r*.

### Formal properties of braille letters

The structural constraints described above make braille an ideal candidate for formal information theory and computational analysis. In this section, we examine the information structure of braille letters using two tools from cognitive modeling: information-theoretic measures (informativeness and cue validity) and linear separability analysis. These tools allow us to ask: Which dots are most informative for distinguishing letters? And can the braille alphabet be perfectly separated based on dot configurations alone?

**Fig. 2:**
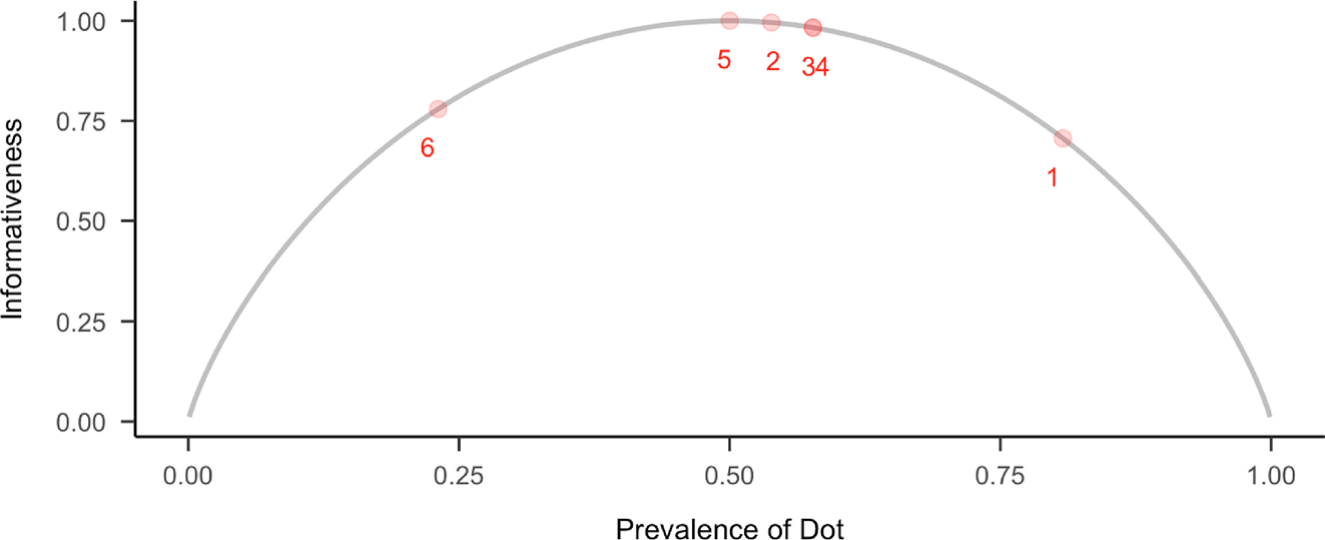
Informativeness as a function of feature; the x-axis represents the proportion of letters containing a given feature, and the y-axis shows the Informativeness (binary entropy). The gray curve depicts the theoretical binary entropy function, and the red points represent the actual prevalence and informativeness of each of the six braille dot positions, labeled 1 through 6: Dot 1: 0.706, Dot 2: 0.996, Dot 3: 0.983, Dot 4: 0.983, Dot 5: 1.000, Dot 6: 0.779

Informativeness quantifies how well a dot divides the set of letters. A dot that appears in half the letters maximizes informativeness, whereas one that is nearly universal or extremely rare contributes little to discrimination. To assess the informativeness of elements for distinguishing letters within the braille system, we applied a calculation method recently used by Kim et al. (2025) for phoneme classification. Specifically, we adapted this approach to dot positions to identify those that contribute most to letter differentiation in braille. This measure is based on Shannon (1948) entropy, which captures the uncertainty associated with the distribution of a feature across letters. It is calculated as:

1 $$H( p ) = - p{\mathrm{log}}_{{2}} ( p ){-}( {1 - p})\;{\mathrm{log}}_{2} ( {1 - p}),$$

where *p* represents the proportion of letters possessing a given feature. The function reaches its maximum when the feature is present in half of the letters (*p* = 0.5), reflecting the greatest level of uncertainty and, thus, the highest informativeness. The prevalence of a feature and its informativeness have an inverted u-shaped relationship. Figure 2 plots informativeness values for each dot in the braille cell.

**Fig. 3:**
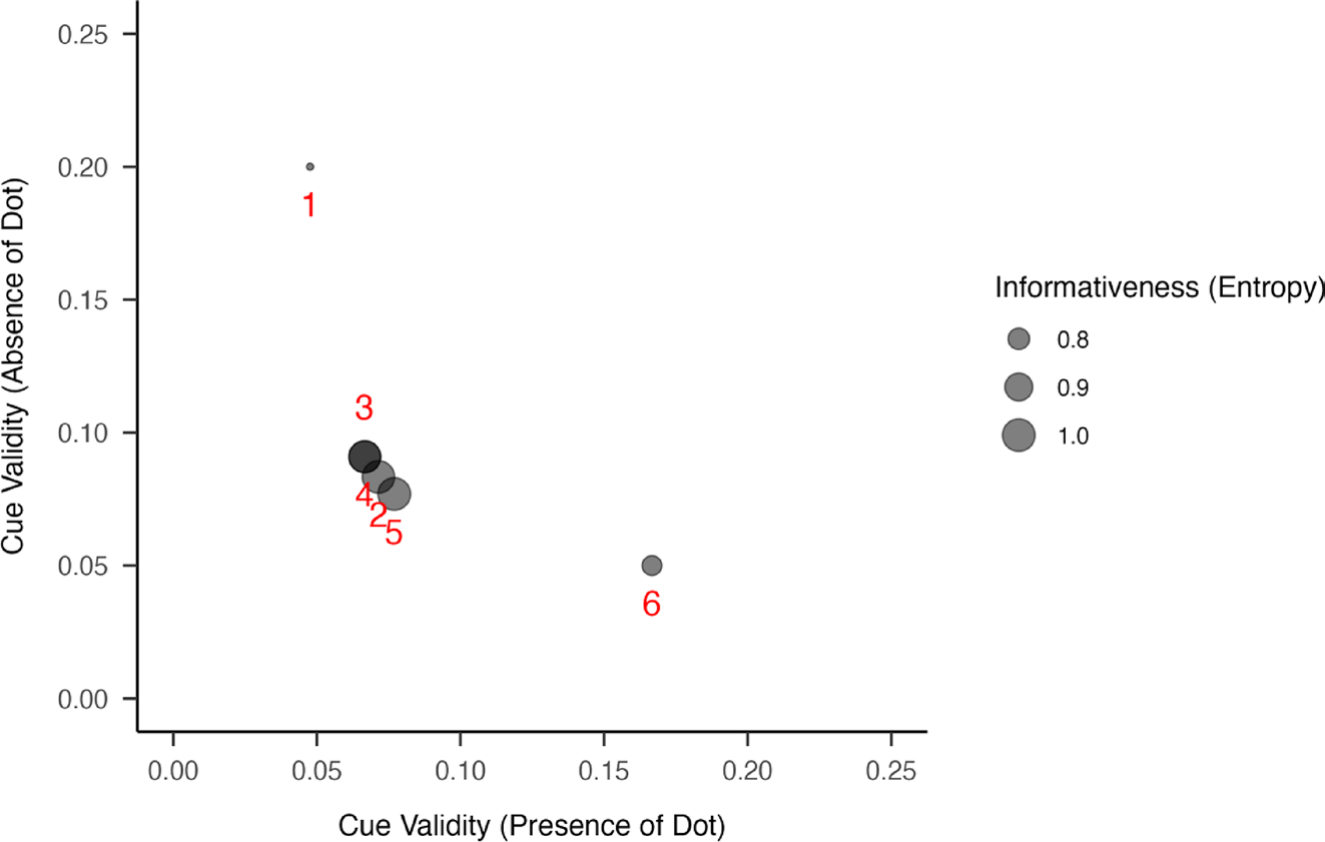
Scatterplot of cue validity for the presence and absence of each of the six braille dot positions. Each point represents a dot, labeled 1 through 6. The x-axis shows the cue validity of the dot’s presence *p*(*l|d*), while the y-axis shows cue validity for its absence *p*(*l|*~ *d*). Dot size reflects the informativeness of each feature, computed as binary. Note how the presence and absence can contribute unequally to categorization, with certain dots being informative primarily in one state

In addition, cue validity (Rosch & Mervis, 1975; see also Beach, 1964; Reed, 1972) provides a complementary probabilistic view: It estimates the likelihood of a specific letter given the presence or absence of a particular dot (i.e., how diagnostic a given dot is for identifying a particular letter). Each braille dot is a binary feature whose presence or absence provides probabilistic evidence for letter identity. The presence-form cue validity, formally expressed as *p*(*l∣d*), is the probability of letter *l* given that a specific dot *d* is raised, whereas the absence form *p*(*l|*~ *d*) is the probability of *l* given that dot’s absence. These two forms can have asymmetric diagnostic value: A dot’s presence might be far more informative than its absence (or vice versa) depending on how that dot is distributed across letters. For example, if a particular braille dot is present in only a few letters, that dot strongly cues those letters—there is a high *p*(*l|d*), while its absence yields little clue: There is a low *p*(*l|*~ *d*), since many letters lack it. Figure 3 shows the scatterplot for the presence cue validity vs the absence cue validity. Because the first dot is present in many letters, its absence is particularly informative; the opposite is the case for dot 6. Asymmetries between presence and absence cue validity reveal how individual dots can offer strong cues for some letters but not others (see Fig. 3).

Importantly, structural informativeness does not necessarily translate into perceptual discriminability. In classification problems, classes are linearly separable if a hyperplane exists that perfectly separates all data points of one class from those of another. For binary inputs (such as braille dot patterns), one can test linear separability using a linear classifier, such as a perceptron. In simple terms, this analysis tests whether the pattern of raised dots alone provides enough information for a simple model to tell each letter apart, without the need for more complex layers or learning mechanisms. In 6-dot braille, there are 2^6^ = 64 possible combinations (63 if we do not consider an empty cell). When considering only the 26 letters of the alphabet, the problem becomes tractable: One can empirically assess whether a linear classifier can distinguish these 26 patterns without error, thereby determining whether the braille alphabet is linearly separable in a six-dimensional binary feature space defined by dot presence or absence.

To test this, we implemented a simple perceptron (a two-layer neural network consisting of an input and output layer, with no hidden layer). In this architecture, each input unit encoded the binary state of one braille dot (6 units total), and each output unit corresponded to one letter in the alphabet (26 units). Network weights were initialized randomly from −0.3 to 0.3 and trained using the delta rule with a sigmoid activation function and a learning rate of 0.4. For each training pattern, the net input to each output unit was the weighted sum of its inputs, which was then passed through the logistic sigmoid to produce an activation. The error signal for each output was computed as the product of the prediction error and the derivative of the activation function. Weights were updated after each pattern presentation, with a small momentum term to stabilize learning. Training proceeded for up to 5000 epochs, or until no further improvement was observed, with patterns presented in random order each epoch.

**Fig. 4:**
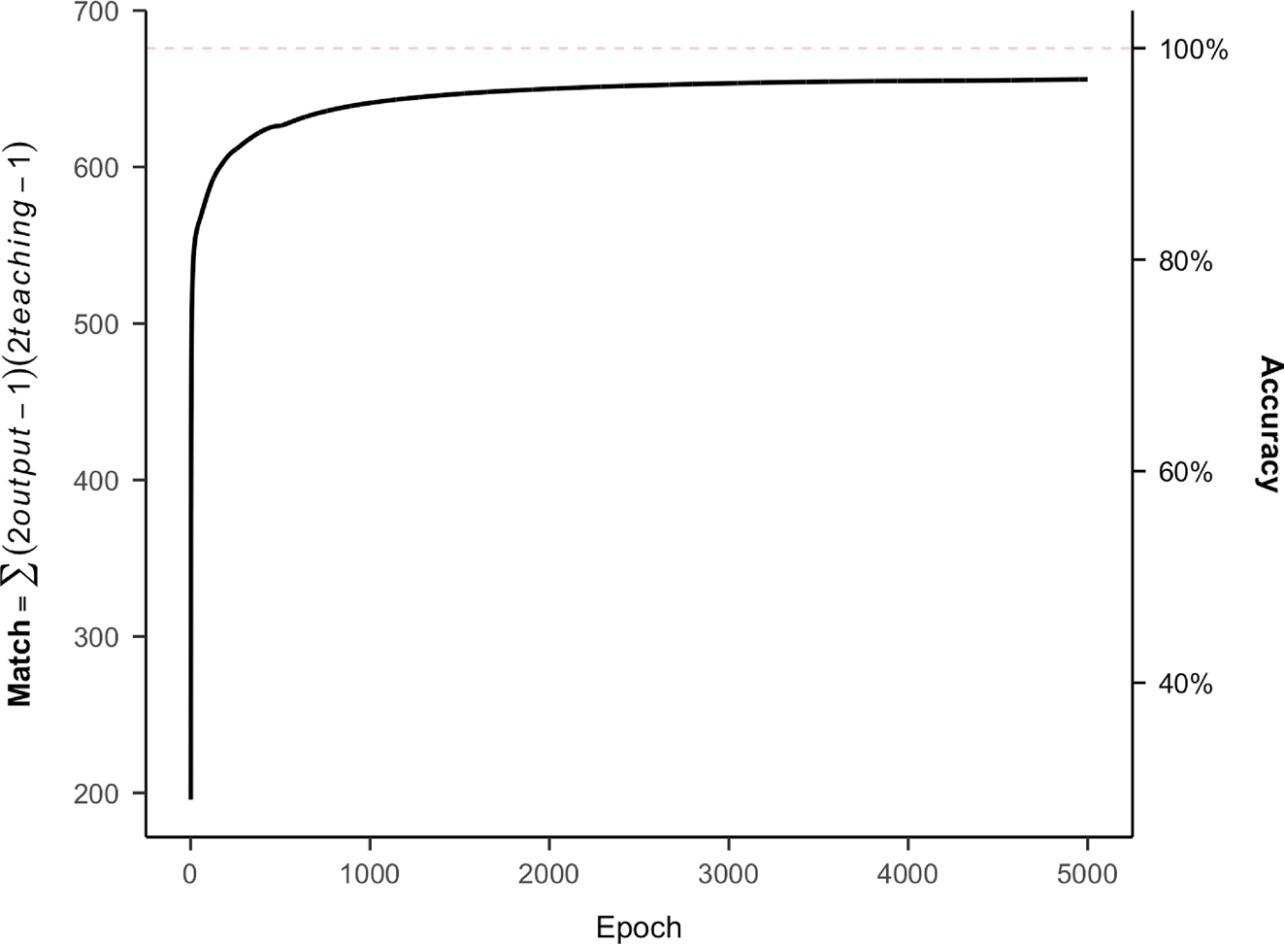
Training performance of the linear neural network across 5000 epochs. The x-axis shows the number of training iterations, and the y-axis indicates the match between the desired output (teaching patterns) and the model’s activation in the output layer (out of a maximum possible value of 676 [26 letters × 26 output units]). This is an illustrative run of the model, as performance can vary slightly due to starting values; note that in none of the many runs of the model, did we get to full identification of the characters

Because the model contained no hidden layer, it could only learn linear decision boundaries. Regardless of the number of epochs or the order of training patterns, the model consistently failed to classify four letters correctly: *f **, g **, o *, and *p * (see Fig. 4). This outcome indicates that tactile letter recognition must rely on more complex internal representations beyond simple dot-based features; the similarity matrix presented in the next section aims to uncover what are the character groupings and the features that underlie braille letter recognition. Given that this simple linear classifier (i.e., a perceptron with no hidden layer) failed consistently on a small subset of letters (f, g, o, p; see Fig. 4), we conclude that perfect identification of braille letters cannot rely solely on fixed linear combinations of individual dot positions. Instead, successful letter recognition likely depends on higher-order feature interactions or rapid sequential integration during tactile exploration.

### The role of perception in braille recognition

**Fig. 5:**
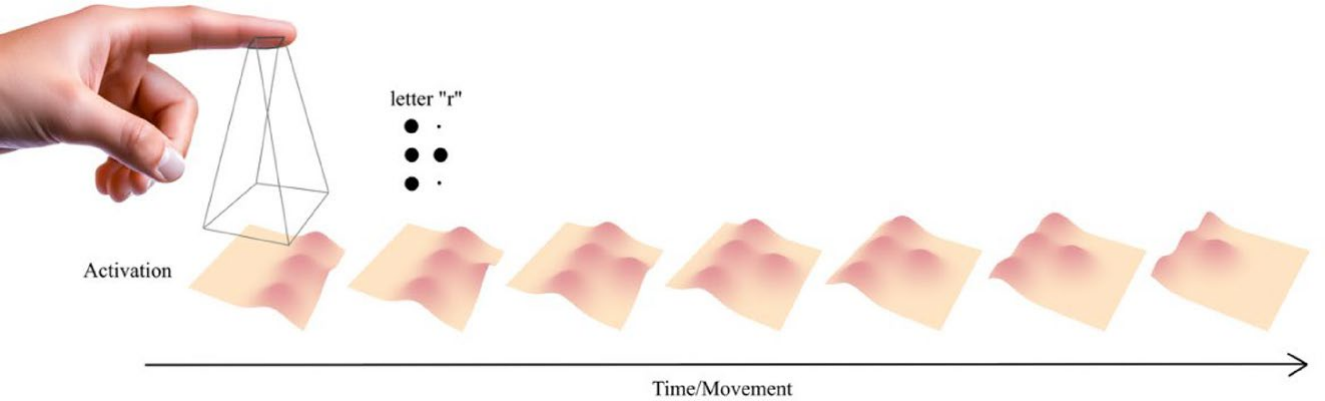
Illustration of dynamic skin deformation during braille reading. As the fingertip moves across a braille cell, the skin undergoes a series of deformations due to contact with raised dots, leading to activation unfolding over time as the finger moves

While formal analyses offer insight into the structural properties of braille, they do not constitute process models. Most notably, they fail to account for the sensory motor dynamics of tactile reading. Braille is read not by passively perceiving static dot arrays, but instead, by actively sliding the fingertip across the page. As the finger moves, raised dots deform the skin in a time-locked sequence, creating a temporally unfolding tactile trace. Figure 5 illustrates this process. As the fingertip glides over a braille cell (e.g., the letter *r *), localized contact with raised dots produces a sequence of skin deformations. These deformations activate mechanoreceptors over time, forming a perceptual stream that must be integrated to extract the letter’s identity. This dynamic and sequential mode of perception introduces patterns of similarity and dissimilarity not captured by static spatial metrics such as dot overlap and has a serial component as depicted in Fig. 5. This seriality cannot be captured by the formal analysis of dots, and hence, empirical research is in order.

This raises a fundamental question: What are the perceptual units of braille reading? Early theories emphasized holistic or “outline” recognition (Nolan & Kederis, 1969 proposed that braille characters are perceived as shapes based on perceptual processes akin to good continuation, such that *l * is perceived as a continuous vertical line ), whereas later studies showed that subtle variations in spacing and configuration impair recognition, suggesting a finer-grained, dot-level encoding (Millar, 1977, 1985). More recent reports (Graven, 2015) point to individual differences: Some readers describe relying on local dot features, others on global configurations. However, subjective reports are inherently limited. The processes that transform spatiotemporal skin input into letter representations are not readily accessible to conscious awareness, and introspection often fails to capture the underlying mechanisms.

### Goals of the study

Research on letter recognition in the visual modality has long relied on empirically derived similarity matrices (e.g., Fiset et al., 2008; Mueller & Weidemann, 2012; Simpson et al., 2012) to design experiments and constrain models. However, the tactile modality lacks an equivalent resource. Our primary goal is to fill that gap by creating an empirically grounded perceptual similarity matrix for braille letters in a sample of fluent blind readers. This matrix is intended as a use-inspired basic resource: It advances theory while being directly usable to control confusability in experiments and to guide instructional sequencing and evaluation in braille literacy.

A second goal is to link structural properties of the braille cell (e.g., entropy-based informativeness and cue validity of individual dots; linear separability of dot patterns) to observed perceptual similarity, clarifying where formal features do, or do not, predict human discriminability. This allows us to test whether constraints inferred from dot distributions align with constraints that emerge in touch.

To meet these goals, we combine formal analyses of dot features with a same–different task testing all 26 basic letter pairs in braille with congenitally blind participants. In this task, readers judge whether two braille letters are the same or different—a method that yields fine-grained measures of tactile confusability and enables the construction of a perceptual similarity matrix grounded in real-world experience. This study may be useful in (a) experimental design (e.g., when matching or varying letter similarity), (b) instructional contexts (sequencing letters from different clusters to reduce early confusions), (c) technology evaluation (as benchmarks when letters differ by sparsely informative dots), and (d) theoretical models of tactile word recognition, by presenting empirically grounded similarity spaces for model testing.

## Method

Materials and scripts for data analyses can be found in the OSF repository: https://osf.io/98ek7/?view_only=89a6a4203f6b4450ae93e4d0033635e3.

### Apparatus

We used Baciero et al.’s (2023b) TouchScope system to control the presentation timing of braille stimuli while maintaining the needed movement of the fingertips across the braille letters from left to right. This system consists of a refreshable braille display (i.e., Active Braille, Help Tech, 2018; see Saladino, 2019) placed on a motorized platform that moves the display horizontally at a set speed and distance (see Baciero et al., 2023b, for photographs and assembly instructions of the apparatus). Thus, with this system, we slid the braille letters on the braille display against participants’ fingers instead of having participants move their fingers from left to right to perceive the letters; this allowed us to have enough timing control to be able to record response times in the same/different task. In addition, we used 3D stickers to indicate the area where the braille letters would appear, serving as reference points (start and end). The display was connected via USB to a MacOS; a shell script was used to present the stimuli on the braille display, as well as to record the participant’s responses.

### Participants

Twenty-four blind, fluent braille readers (13 males; mean age = 37.3 years, range = 19–59), all native Spanish speakers, participated in the study. Most participants had been diagnosed with severe visual impairment or congenital blindness and began Braille instruction early in life (between ages 5 and 8). Two participants had residual vision at birth and lost their sight during childhood, starting braille instruction later (at ages 11 and 16, respectively). Participants were recruited with the support of the National Organization of Spanish Blind People (ONCE, by its Spanish acronym). All gave informed consent prior to participation and received financial compensation (€15 per session). With this sample size, we wanted to ensure each pair of different letters was observed a minimum of 20 times (considering pairs containing the same two different letters in the opposite order as being different pairs (e.g., *a **b * was a different pair than *b ** a *), and taking into account that some trials may be lost in data cleaning).

### Materials

The study used all possible 2-letter combinations (n = 676) pairs. Out of those pairs, 26 were formed by the same two letters (e.g., *a **a *) and 650 formed by two different letters (e.g., *a **b *). To have the same number of trials per condition (i.e., same and different), we created five different lists of pairs, each of them with 130 same pairs, and 130 different pairs. All participants were exposed to all the lists in two (3 + 2 lists) or three (2 + 2 + 1 lists) experimental sessions. The order of presentation of the target trials was randomized for each participant, and we included six practice trials at the beginning of each list.

### Procedure

The experiment took place individually in a quiet room. Participants were instructed to place their index fingertip of their dominant hand on the start position (next to the first 3D sticker) and let the braille display slide against it. After sensing the stimuli, they were asked to perform a same/different judgment task, in which they had to classify the two letters presented per trial, as quick and accurate as possible, as being the same two letters (e.g., *a **a *) or two different letters (e.g., *a **b *). Responses were made by pressing either **M** or **N**, respectively, on the computer keyboard with their non-dominant hand—note that these two keys are contiguous on the Spanish keyboards we used, allowing the use of two fingers of the same hand at no motor cost. The braille display moved for approximately 5 cm at 173 mm/s (40 mm/rev × 260 rpm/60). This speed was selected after a testing session with an expert braille reader, as well as taking into consideration our own experience using the device. After moving said distance, the display stopped until participants responded and reset its position during the 1.3 s inter-stimulus interval (ISI). Each list took around 30 min to complete.

## Results

We present behavioral results from the same–different task involving all braille letter pairs. We first report overall performance and construct dissimilarity matrices from both accuracy and response time (RT) data. Accuracy reflects confusability—how often one letter is mistaken for another—and informs us about representational overlap in perceptual space. RT, in contrast, reflects processing efficiency. We then apply hierarchical clustering to identify perceptual groupings of letters. Finally, we analyze “same” trials to evaluate the role of dot informativeness in individual letter processing.

Scripts for data wrangling and cleaning (based on criteria established prior to data collection; see Gomez et al., 2017) are available at https://osf.io/98ek7/. Response times and accuracy were recorded for each trial. RTs shorter than 0.2 s or longer than 8 s were excluded (0.47% of the data), as were error trials in the RT analysis (5.32% of the data).

Table 1 shows the mean accuracy and mean response times per trial type (same or different) averaged across participants. Note that the method that we employed to create the similarity matrix is based on data obtained under speeded presentation of the stimuli to slightly increase the number of errors made by participants; this is an approach typically used in previous studies examining letter similarity (e.g., Mueller & Weidemann, 2012; Wiley et al., 2016). In addition to constructing similarity matrices for braille letters, we carried out a hierarchical clustering analysis to visualize the groups of letters that are perceived to be similar to each other and hence different from others.

**Table 1:** Mean accuracy (proportion) and RT (for correct and error responses, in ms) across participants per trial type

Trial type	Accuracy	RT_correct_ (ms)	RT_error_ (ms)
Same	0.927	1537	1724
Different	0.966	1548	1785

For the accuracy data, responses were aggregated across participants into a matrix in which each cell reflected the proportion of correct responses for a given letter pair (row × column). Hence, higher values indicated greater dissimilarity between the two letters, with values approaching 1 reflecting easier discrimination (i.e., lower perceptual similarity). Before averaging the matrices, we verified whether perceptual similarity judgments were directionally symmetric (e.g., the pair *a * → *z * might differ from *z * → *a * which could affect our ability to treat the matrix as symmetric). To assess this, we computed Bayes factors (with the Bayes Factor package; Morey et al., 2024) comparing the upper and lower triangles of the matrix (excluding the diagonal, of course) using a paired-sample *t* test. The results, *t*(324) = 0.637, BF₀₁ = 13.14, provided substantial evidence in favor of the null hypothesis of symmetry. Accordingly, we averaged the upper and lower triangles to create a single, symmetric dissimilarity matrix (see Table 2).

**Table 2:** Dissimilarity matrix from accuracy data

	a	b	c	d	e	f	g	h	i	j	k	L	m	n	o	p	q	r	s	t	u	v	w	x	y	z
a	**0.981**	1	0.905	0.959	0.963	0.981	1	1	0.938	1	0.943	0.979	0.94	1	0.962	0.98	1	0.94	1	1	0.979	1	0.98	0.959	1	0.962
b	1	**0.945**	0.945	0.979	0.98	0.961	0.866	0.917	0.983	0.979	0.96	0.808	0.979	1	0.962	0.979	0.981	1	0.961	1	1	0.982	0.982	0.961	1	0.981
c	0.905	0.945	**0.976**	0.98	1	1	1	0.979	0.981	0.963	0.98	0.981	1	1	1	1	0.98	1	1	1	1	0.981	0.98	0.982	0.961	0.96
d	0.959	0.979	0.98	**0.959**	0.9	0.957	0.96	0.9	1	1	1	1	1	0.981	0.943	1	1	0.981	1	0.981	1	0.981	0.98	1	0.98	1
e	0.963	0.98	1	0.9	**0.948**	1	1	0.96	1	0.981	1	0.963	1	1	1	1	1	0.979	0.961	1	1	0.961	0.98	0.982	1	1
f	0.981	0.961	1	0.957	1	**0.913**	0.824	0.901	0.981	0.921	1	0.96	0.979	1	0.981	0.96	0.96	1	0.961	0.924	1	1	0.982	0.982	1	0.982
g	1	0.866	1	0.96	1	0.824	**0.917**	0.942	1	0.944	1	1	1	1	1	0.963	0.94	0.959	0.96	1	0.94	0.959	0.945	1	0.979	1
h	1	0.917	0.979	0.9	0.96	0.901	0.942	**0.948**	1	0.914	1	1	1	1	0.982	1	0.962	0.882	1	1	1	0.922	0.959	1	0.979	1
i	0.938	0.983	0.981	1	1	0.981	1	1	**0.924**	0.745	1	0.982	1	1	1	0.982	0.979	1	0.92	0.98	0.982	1	0.98	0.961	0.961	1
j	1	0.979	0.963	1	0.981	0.921	0.944	0.914	0.745	**0.939**	0.98	0.982	0.961	1	0.96	0.962	1	1	0.979	0.941	0.982	0.98	0.96	0.98	1	1
k	0.943	0.96	0.98	1	1	1	1	1	1	0.98	**0.939**	0.98	0.94	0.98	0.961	0.981	1	1	1	1	0.867	1	1	0.941	0.982	0.98
l	0.979	0.808	0.981	1	0.963	0.96	1	1	0.982	0.982	0.98	**0.945**	1	1	1	0.94	0.94	0.882	0.981	0.926	0.96	0.885	0.98	0.981	0.98	1
m	0.94	0.979	1	1	1	0.979	1	1	1	0.961	0.94	1	**0.932**	0.979	1	0.942	0.979	0.945	1	1	0.9	0.959	0.981	0.778	0.963	1
n	1	1	1	0.981	1	1	1	1	1	1	0.98	1	0.979	**0.882**	0.701	0.98	0.96	0.981	0.96	0.981	1	1	0.904	0.981	0.704	0.734
o	0.962	0.962	1	0.943	1	0.981	1	0.982	1	0.96	0.961	1	1	0.701	**0.917**	1	1	0.982	0.98	0.979	0.98	1	0.963	1	0.979	0.765
p	0.98	0.979	1	1	1	0.96	0.963	1	0.982	0.962	0.981	0.94	0.942	0.98	1	**0.889**	0.88	0.877	0.908	0.923	0.981	0.96	0.938	1	0.94	1
q	1	0.981	0.98	1	1	0.96	0.94	0.962	0.979	1	1	0.94	0.979	0.96	1	0.88	**0.893**	0.863	0.94	0.824	1	0.937	0.896	1	0.938	0.982
r	0.94	1	1	0.981	0.979	1	0.959	0.882	1	1	1	0.882	0.945	0.981	0.982	0.877	0.863	**0.888**	1	0.918	0.98	0.883	0.86	1	0.963	0.919
s	1	0.961	1	1	0.961	0.961	0.96	1	0.92	0.979	1	0.981	1	0.96	0.98	0.908	0.94	1	**0.946**	0.711	0.981	0.979	0.959	0.965	0.981	0.981
t	1	1	1	0.981	1	0.924	1	1	0.98	0.941	1	0.926	1	0.981	0.979	0.923	0.824	0.918	0.711	**0.907**	0.981	0.981	0.845	0.98	1	1
u	0.979	1	1	1	1	1	0.94	1	0.982	0.982	0.867	0.96	0.9	1	0.98	0.981	1	0.98	0.981	0.981	**0.945**	0.981	0.981	0.869	0.982	0.96
v	1	0.982	0.981	0.981	0.961	1	0.959	0.922	1	0.98	1	0.885	0.959	1	1	0.96	0.937	0.883	0.979	0.981	0.981	**0.943**	1	1	0.96	1
w	0.98	0.982	0.98	0.98	0.98	0.982	0.945	0.959	0.98	0.96	1	0.98	0.981	0.904	0.963	0.938	0.896	0.86	0.959	0.845	0.981	1	**0.910**	1	0.98	0.94
x	0.959	0.961	0.982	1	0.982	0.982	1	1	0.961	0.98	0.941	0.981	0.778	0.981	1	1	1	1	0.965	0.98	0.869	1	1	**0.936**	0.959	0.979
y	1	1	0.961	0.98	1	1	0.979	0.979	0.961	1	0.982	0.98	0.963	0.704	0.979	0.94	0.938	0.963	0.981	1	0.982	0.96	0.98	0.959	**0.879**	0.783
z	0.962	0.981	0.96	1	1	0.982	1	1	1	1	0.98	1	1	0.734	0.765	1	0.982	0.919	0.981	1	0.96	1	0.94	0.979	0.783	**0.897**

The values represent the average accuracy across all participants

Reaction times were rescaled within participants by dividing each RT by that participant’s mean RT so that the overall average discrimination time for each participant was equal to 1. This normalization step removes overall speed differences between participants and isolates item difficulty at the letter pair level. For each letter pair, the rescaled RTs were then averaged across participants into a matrix in which each cell reflected the median adjusted RT for the corresponding row–column pair (see also Courrieu et al., 2004; Wiley et al., 2016). We assessed the symmetry of the matrix and found no significant difference between the upper and lower triangles, t(324) = –1.96, BF₀₁ = 2.43, providing anecdotal support for the null hypothesis. Although the evidence was anecdotal, it still justifies symmetrizing the matrix for practical modeling purposes. The matrix was therefore symmetrized by averaging corresponding upper and lower triangle values. Finally, we converted the matrix into a dissimilarity matrix by taking the reciprocal of each cell value, such that higher values reflected greater dissimilarity (see Table 3).

**Table 3:** Dissimilarity matrix from RT data

	a	b	c	d	e	f	g	h	i	j	k	l	m	n	o	p	q	r	s	t	u	v	w	x	y	z
a	**1.046**	1.054	0.907	1.029	1.047	0.955	1.002	1.038	0.962	1.045	0.998	0.982	1.005	1.036	1.033	1.054	0.999	1.059	1.014	0.989	1.006	1.048	1.030	1.056	1.043	1.053
b	1.054	**1.031**	0.977	0.992	1.006	0.977	0.993	0.978	0.996	0.995	1.051	0.956	1.082	0.998	1.018	1.002	1.029	0.998	0.997	1.010	1.072	1.045	1.029	1.029	1.029	1.023
c	0.907	0.977	**1.094**	0.971	1.055	1.039	1.043	1.035	1.033	1.013	1.021	1.040	1.060	1.041	1.057	1.047	0.977	1.013	1.072	1.032	1.021	1.024	1.020	1.056	1.092	1.037
d	1.029	0.992	0.971	**1.033**	0.955	0.907	1.009	0.980	1.013	0.953	0.965	0.996	1.028	1.033	1.044	1.042	1.057	1.013	1.031	1.000	1.080	1.027	0.998	1.005	1.013	1.035
e	1.047	1.006	1.055	0.955	**1.04**	1.011	0.961	0.987	0.945	1.005	1.036	1.057	1.034	1.054	0.972	1.072	1.018	1.002	1.030	1.038	1.060	1.070	0.971	1.031	1.098	1.020
f	0.955	0.977	1.039	0.907	1.011	**1.009**	0.920	0.937	1.004	0.919	0.992	0.990	1.049	1.001	1.045	1.059	0.919	0.991	1.028	1.030	1.019	1.046	1.004	1.032	1.019	1.018
g	1.002	0.993	1.043	1.009	0.961	0.920	**1.036**	0.941	1.025	0.987	1.078	0.986	1.025	1.033	1.007	0.956	0.917	0.969	1.022	1.004	1.020	1.037	1.030	1.026	1.021	1.009
h	1.038	0.978	1.035	0.980	0.987	0.937	0.941	**1.013**	0.999	0.883	1.055	1.007	1.061	0.999	1.010	1.009	1.040	0.989	0.989	1.017	1.042	1.001	0.959	1.009	1.036	0.995
i	0.962	0.996	1.033	1.013	0.945	1.004	1.025	0.999	**0.990**	0.901	0.990	1.056	1.006	1.047	1.025	0.993	1.050	1.014	0.965	1.018	1.050	1.057	1.040	1.028	1.033	1.000
j	1.045	0.995	1.013	0.953	1.005	0.919	0.987	0.883	0.901	**0.991**	1.067	1.023	1.103	0.990	1.016	1.086	1.027	1.006	1.006	1.021	1.021	1.015	0.991	1.042	1.026	1.030
k	0.998	1.051	1.021	0.965	1.036	0.992	1.078	1.055	0.990	1.067	**1.016**	1.018	0.942	0.960	0.969	0.997	1.045	0.989	1.009	1.040	0.913	1.020	0.961	0.998	1.004	1.009
l	0.982	0.956	1.040	0.996	1.057	0.990	0.986	1.007	1.056	1.023	1.018	**1.027**	0.992	1.050	1.033	0.945	1.018	0.938	1.024	1.002	0.969	0.983	1.000	1.002	1.006	1.018
m	1.005	1.082	1.060	1.028	1.034	1.049	1.025	1.061	1.006	1.103	0.942	0.992	**1.014**	1.004	0.999	1.004	1.025	1.010	1.007	1.007	0.912	1.033	1.027	0.933	0.968	1.035
n	1.036	0.998	1.041	1.033	1.054	1.001	1.033	0.999	1.047	0.990	0.960	1.050	1.004	**0.964**	0.851	0.953	0.978	0.962	0.997	0.922	0.966	1.011	0.980	0.963	0.934	0.867
o	1.033	1.018	1.057	1.044	0.972	1.045	1.007	1.010	1.025	1.016	0.969	1.033	0.999	0.851	**0.981**	1.008	0.963	0.984	1.013	1.017	0.990	1.028	0.941	0.969	0.943	0.776
p	1.054	1.002	1.047	1.042	1.072	1.059	0.956	1.009	0.993	1.086	0.997	0.945	1.004	0.953	1.008	**0.974**	0.875	0.975	0.958	0.928	1.053	0.934	0.976	0.960	0.986	1.006
q	0.999	1.029	0.977	1.057	1.018	0.919	0.917	1.040	1.050	1.027	1.045	1.018	1.025	0.978	0.963	0.875	**0.948**	0.867	0.938	0.947	1.025	1.017	0.927	0.970	0.971	0.956
r	1.059	0.998	1.013	1.013	1.002	0.991	0.969	0.989	1.014	1.006	0.989	0.938	1.010	0.962	0.984	0.975	0.867	**0.976**	1.015	0.928	1.013	0.906	0.847	1.044	0.963	0.953
s	1.014	0.997	1.072	1.031	1.030	1.028	1.022	0.989	0.965	1.006	1.009	1.024	1.007	0.997	1.013	0.958	0.938	1.015	**0.970**	0.893	0.993	1.032	0.939	1.047	1.016	1.034
t	0.989	1.010	1.032	1.000	1.038	1.030	1.004	1.017	1.018	1.021	1.040	1.002	1.007	0.922	1.017	0.928	0.947	0.928	0.893	**0.978**	0.980	0.978	0.953	1.045	1.002	1.024
u	1.006	1.072	1.021	1.080	1.060	1.019	1.020	1.042	1.050	1.021	0.913	0.969	0.912	0.966	0.990	1.053	1.025	1.013	0.993	0.980	**1.004**	0.950	1.003	0.871	1.021	0.993
v	1.048	1.045	1.024	1.027	1.070	1.046	1.037	1.001	1.057	1.015	1.020	0.983	1.033	1.011	1.028	0.934	1.017	0.906	1.032	0.978	0.950	**1.001**	0.951	0.991	0.976	0.996
w	1.030	1.029	1.020	0.998	0.971	1.004	1.030	0.959	1.040	0.991	0.961	1.000	1.027	0.980	0.941	0.976	0.927	0.847	0.939	0.953	1.003	0.951	**0.931**	1.013	0.945	0.912
x	1.056	1.029	1.056	1.005	1.031	1.032	1.026	1.009	1.028	1.042	0.998	1.002	0.933	0.963	0.969	0.960	0.970	1.044	1.047	1.045	0.871	0.991	1.013	**1.031**	0.900	0.917
y	1.043	1.029	1.092	1.013	1.098	1.019	1.021	1.036	1.033	1.026	1.004	1.006	0.968	0.934	0.943	0.986	0.971	0.963	1.016	1.002	1.021	0.976	0.945	0.900	**0.957**	0.896
z	1.053	1.023	1.037	1.035	1.020	1.018	1.009	0.995	1.000	1.030	1.009	1.018	1.035	0.867	0.776	1.006	0.956	0.953	1.034	1.024	0.993	0.996	0.912	0.917	0.896	**0.958**

The values represent the average RT across all participants

**Fig. 6:**
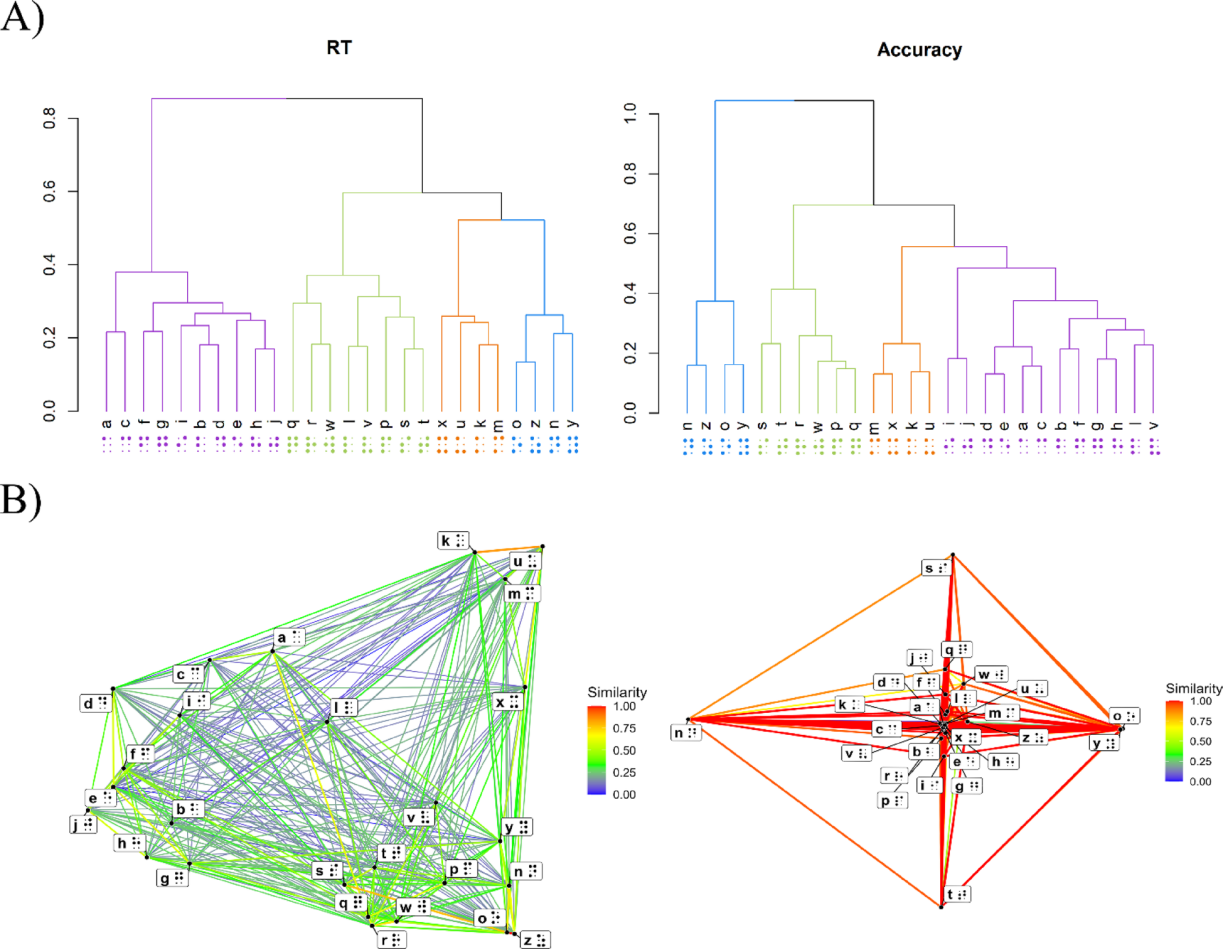
Panel A shows the hierarchical clustering dendrograms based on response times (left) and accuracy (right). The height of the connecting lines reflects dissimilarity, with lower branches indicating greater similarity between letters. Clusters are color coded. Panel B presents a network visualization of letter similarities derived from RT (left) and accuracy (right) data. Line color reflects similarity strength (red = high similarity; blue = low similarity)

To carry out the hierarchical cluster analyses and visualize the letter groupings based on their similarity, we used the stats package in R (R Core Team, 2024). The dissimilarity matrices were turned into distance objects (matrices) using the Euclidean method. Hierarchical clustering was then carried out using Ward’s method (see Murtagh & Legendre, 2011) which identified the strongest clustering structure. Panel A of Fig. 6 shows the resulting dendrograms for accuracy and RT, which provide an overview of the similarity and differences among braille letters. For comparison purposes, cluster colors are held constant between the two dendrograms.

To examine whether the structure of the similarity patterns derived from accuracy and reaction time measures was comparable, we computed a Spearman correlation between the upper triangles of the accuracy-based similarity matrix and the RT-based dissimilarity matrix. The correlation was moderate and positive, *r* = 0.43, *p* < 0.001 (see Appendix A for the scatterplot), indicating that letter pairs that were more difficult to discriminate (i.e., lower accuracy) also tended to elicit longer reaction times. This relationship suggests that both measures capture a common underlying structure of perceptual similarity among Braille letters.

The dendrograms in Fig. 6 revealed four stable clusters of letters, largely corresponding to shared dot patterns. These clusters provide a structured view of the perceptual similarity among braille letters, derived from both accuracy and RT data. Note that the RT data provide a more nuanced view of similarity as the space is quite compressed in the case of accuracy (there are a lot of cells with ceiling level performance of accuracy = 1.0). For this reason, and to assess how well the hierarchical clustering captured the underlying perceptual structure of the braille letters, we computed the cophenetic correlation between the dendrogram and the original 1/RT dissimilarity matrix. The cophenetic correlation quantifies the degree to which the pairwise distances implied by the dendrogram preserve the original dissimilarities among stimuli (Sokal & Rohlf, 1962). For 1/RT, the dendrogram achieved a cophenetic correlation of *r* = 0.67, indicating a moderate correspondence between the hierarchical structure and the raw reaction time–based dissimilarities. This means that the dendrogram captures the broad organization of the perceptual space, but fine-grained pairwise relationships are represented only approximately.

The results show four clusters of letters: (1) *n *, *z *, *o *, *y * in blue; (2) *s *, *t *, *r *, *w *, *p *, *q * in green; (3) *m *, *x *, *k *, *u *in orange; (4) *i *, *j *, *d *, *e *, *a *, *c *, *b *, *f *, *g *, *h * in purple. Letters *l * and *v * are part of the purple group in the accuracy dendrogram and of the green group in the RT dendrogram. Bootstrap resampling with 1000 iterations (using the pvclust R package; Suzuki & Shimodaira, 2023) provided approximately unbiased (AU) values for each cluster; all clusters achieved AU ≥ 0.90, which the highest AU is 0.97 for the orange cluster.

Interestingly, these clusters relate to some (but not all), of the findings from our formal analyses. For instance, different combinations of presence/absence of dots 2, 3, and 5, which are three of the most informative dots, seem to be key characteristics in differentiating clusters and determining similarity among letters within each cluster. Specifically, the purple group is formed by letters that have absence of dots 3 and 6 ( , i.e., the 10 first letters of the alphabet, being the “base” braille patterns). Notably, the absence of dot 3 separates this group from the rest. The green group is formed by letters that have dots risen in both columns and in every row of the braille cell, and it is the presence of dot 2 that differentiates this group from the other two. Moreover, given that letter *w * is rare in Spanish and that it is a rotation of letter *r *, it could be that a key characteristic of this group is the presence of dots 2 and 3 ( ), which differentiates it from all the other groups. The blue group is also formed by letters that have raised dots in every column and row of the cell, but the combination of the absence of dot 2 and the presence of dots 3 and 5 ( ) is what may explain the similarity among letters in this cluster and their distinction from the other clusters. Finally, the orange group is formed by letters that have the absence of dots 2 and 5, and the presence of dot 3 ( ). Moreover, these clusters also highlight the lesser informativeness of dot 6, as letters differing only in the presence or absence of this dot are perceived as very similar (i.e., *k *, *u *, *m *, *x * [orange group], *n *, *y *, *o *, *z * [blue group], and letters *l * and *v *, which are ambiguous in cluster membership).

These results suggest that letters are recognized based on the specific arrangement of dots. In fact, it has been proposed that dot density within a cell—that is, not only the number of raised dots but also their proximity to one another—plays a key role in letter recognition (see Millar, 2003). It is also worth considering whether sequential access within the braille cell contributes to letter recognition. Although this type of column-wise processing could, in principle, influence discrimination, the similarity structure observed in the current data does not follow a column-based organization. Instead, the key discriminative features span both columns, suggesting that braille letters are processed as integrated tactile configurations rather than as sequentially scanned halves.

### Same trials

“Same” trials, often treated as controls, offer unique insight into perceptual fluency (i.e., the ease with which a single letter’s pattern can be encoded, maintained, and matched). Unlike “different” trials, they do not require discriminating between competing representations. Their contribution is conceptually complementary to similarity-based matrices. While “different” trials are typically used to quantify letter confusability, same trials are equally informative, albeit in a different way. Because they involve matching two physically identical stimuli (e.g., *b ** b *), they do not require fine-grained discrimination between similar patterns but rather tap into the ease with which a letter’s internal structure can be encoded, maintained, and verified. If certain letters consistently elicit faster or more accurate responses than others in same trials, this implies that some configurations are inherently more legible or unambiguous. In this way, “same” trials offer a valuable measure of intrinsic perceptual accessibility, complementing the relational data provided by different trials. They serve as a baseline against which more complex judgments can be interpreted and help isolate letter-specific properties that influence tactile fluency.

To examine these effects, we computed a per-letter informativeness score by averaging the entropy-based informativeness values of the dots that comprise each letter (see Fig. 2). We then plotted these values against mean response time (RT) and accuracy for each letter in same trials. Although accuracy was generally high across letters (M = 96.3%, SD = 3.1%), performance was not uniform.

**Fig. 7:**
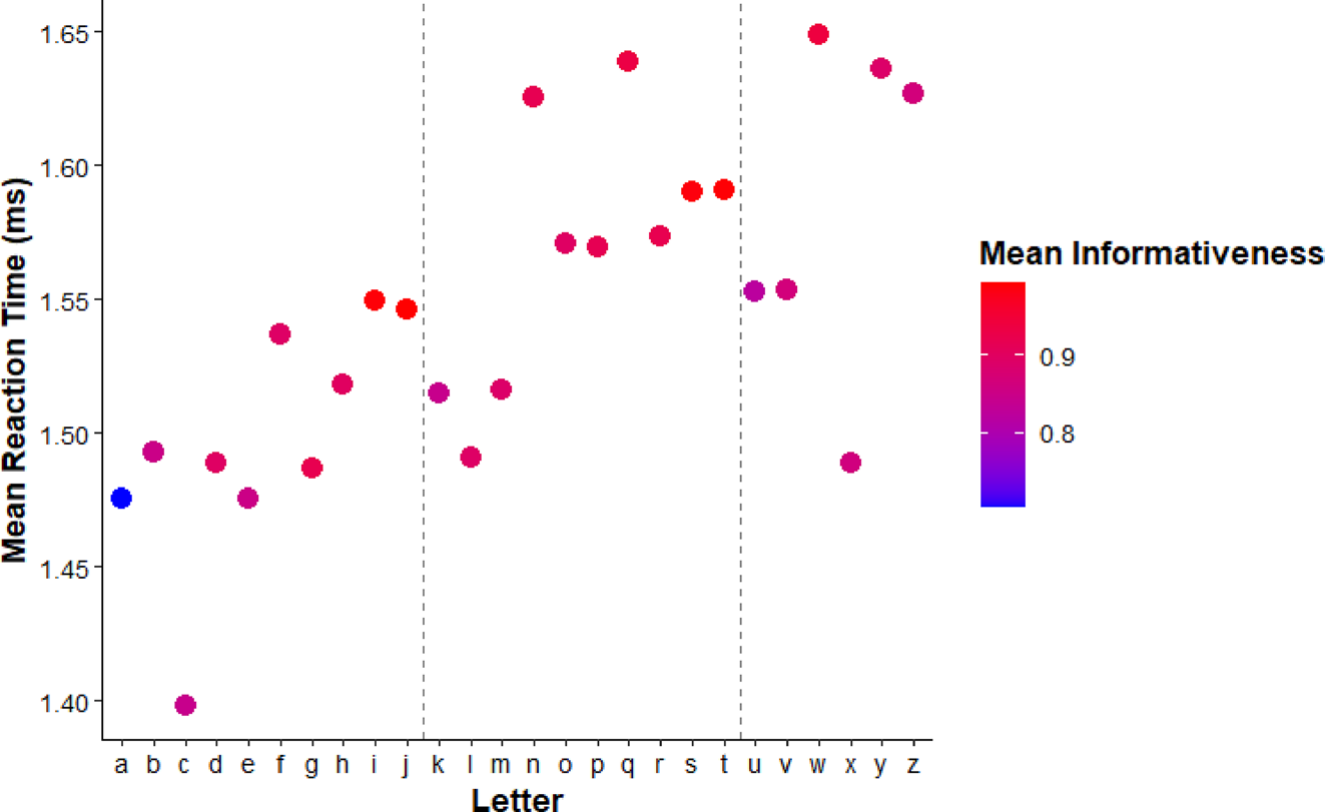
Scatterplot depicting the relationship between the informativeness score of each letter and the mean response time for ‘same’ pairs. Each dot is color coded to represent the mean informativeness of the letter it represents on the x-axis, with low informativeness in blue and high informativeness in red

**Fig. 8:**
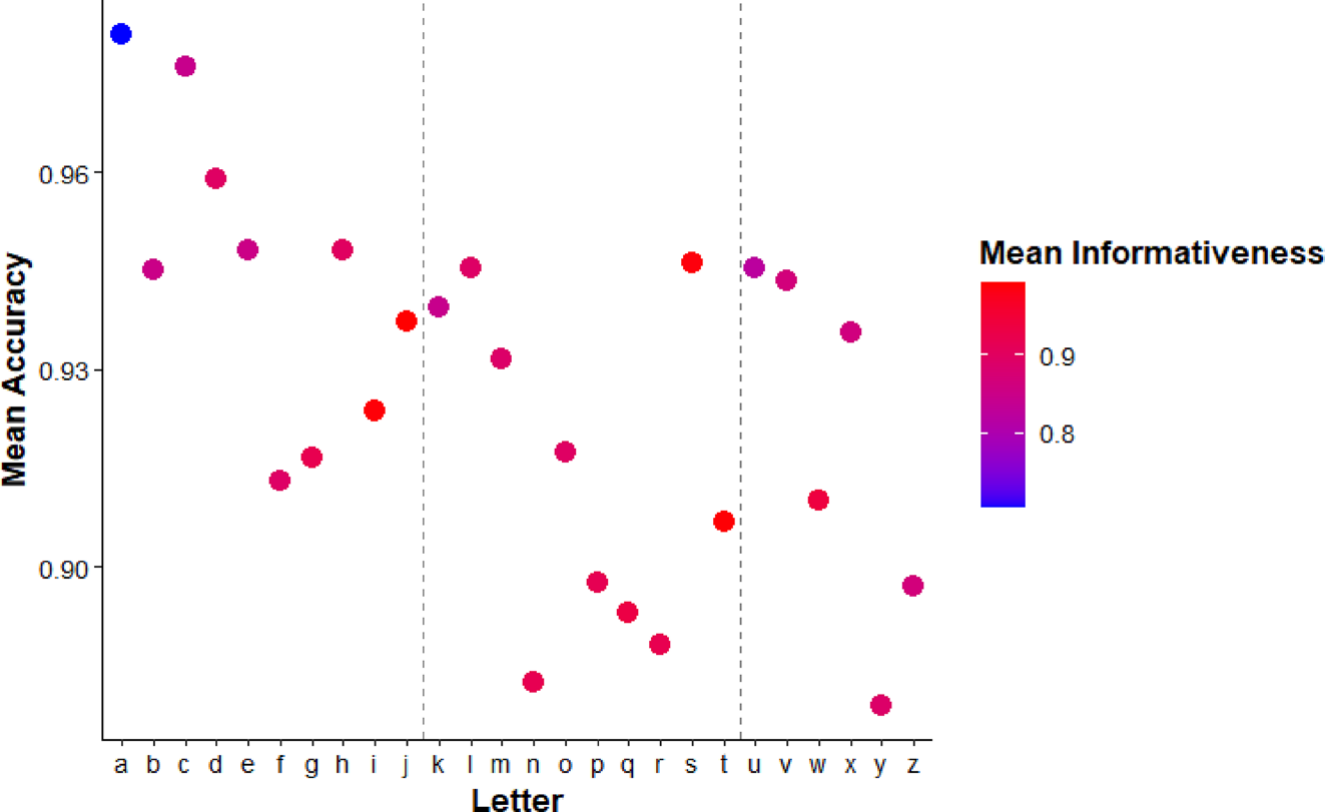
Scatterplot depicting the relationship between the informativeness score of each letter and the mean accuracy for ‘same’ pairs. Each dot is color coded to represent the mean informativeness of the letter it represents on the x-axis, with low informativeness in blue and high informativeness in red

As shown in Fig. 7, which displays the RT per letter, informativeness was robustly associated with response times: Letters with more informative dot configurations were recognized more quickly, with a negative correlation of *r* = –0.43. This suggests that informativeness facilitates encoding—even when only one internal representation must be accessed. In Fig. 8, which displays accuracy, a positive but weaker trend was observed: Informativeness correlated modestly with higher accuracy (*r* = 0.28). Notably, overall accuracy was high (*M* = 96.3%, SD = 3.1%), so variability across letters might be reduced due to near ceiling performance.

Crucially, these effects were not reducible to dot count. For example, some letters with the same number of raised dots yielded markedly different RTs and accuracy depending on which specific dot positions were occupied. This reinforces the notion that informativeness captures perceptual distinctiveness beyond physical complexity.

To illustrate this, letter *x *, despite having four raised dots, was processed more rapidly than many three-dot letters—a striking case where informativeness outweighed dot count. Likewise, letters *d * and *h * both contain three dots, yet *d * was recognized faster, consistent with a more informative dot configuration. These examples highlight how the specific arrangement of dots, not just their number, plays a key role in tactile letter recognition.

**Fig. 9:**
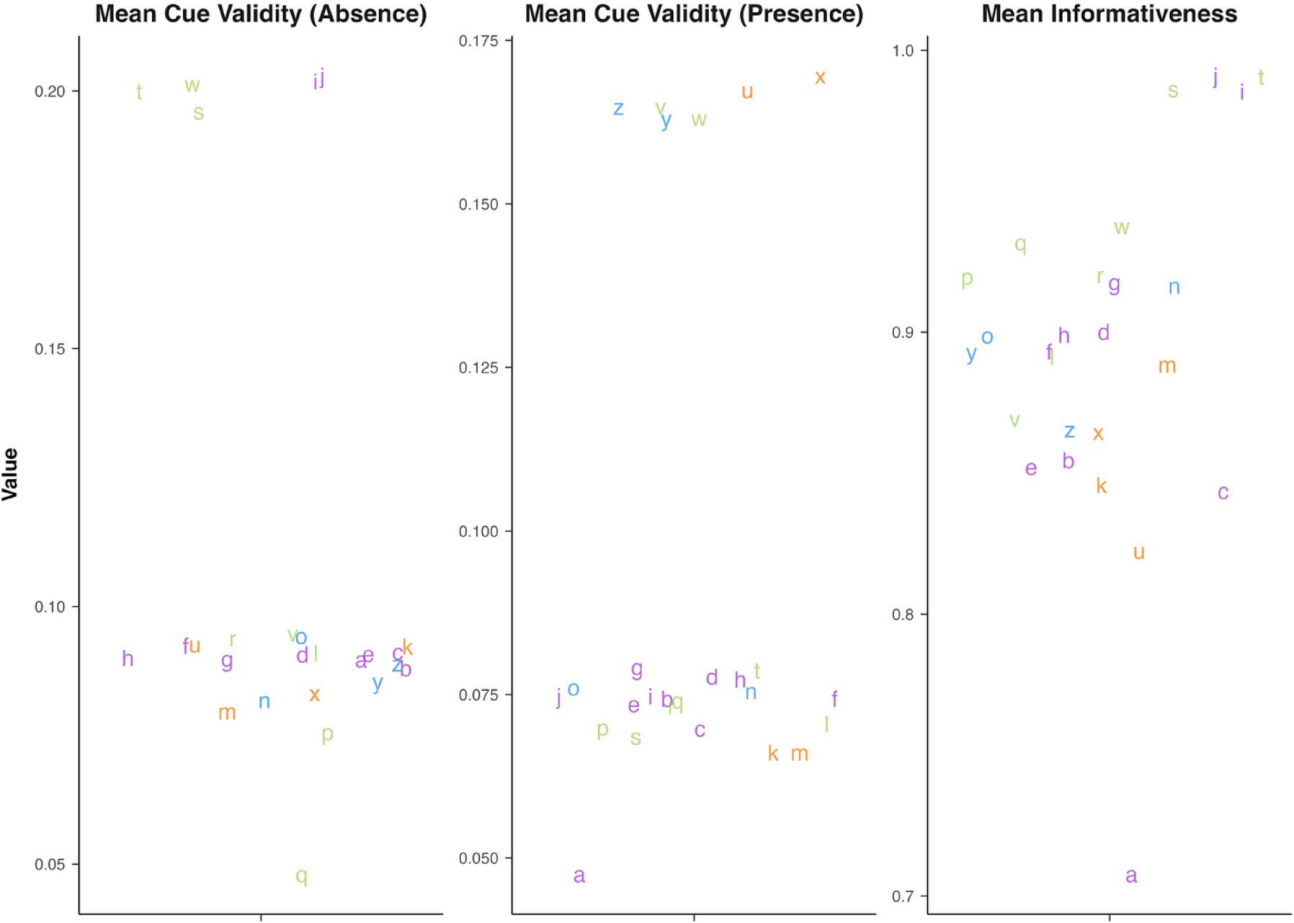
Plot displaying the values of cue validity and informativeness for each of the letters. The values are obtained by averaging the values for each dot within a character. The figure features horizontal jitter so that the letters are visible, and the colors indicate membership in different clusters as obtained in the section above

In addition to these findings, in Fig. 9, we plotted each letter based on three structural metrics (i.e., informativeness, cue validity for presence, and cue validity for absence), with colors reflecting their cluster group from the dendrogram. This visualization showed that these three metrics alone did not group the letters in a way that resembled the dissimilarity-based clusters. In other words, informativeness and cue validity, while helpful for understanding perceptual fluency in same trials, do not fully capture the confusability patterns observed across letters. This suggests that structural properties alone are not sufficient to predict how letters are grouped perceptually, highlighting the importance of combining structural and behavioral data when modeling tactile letter recognition.

**Fig. 10:**
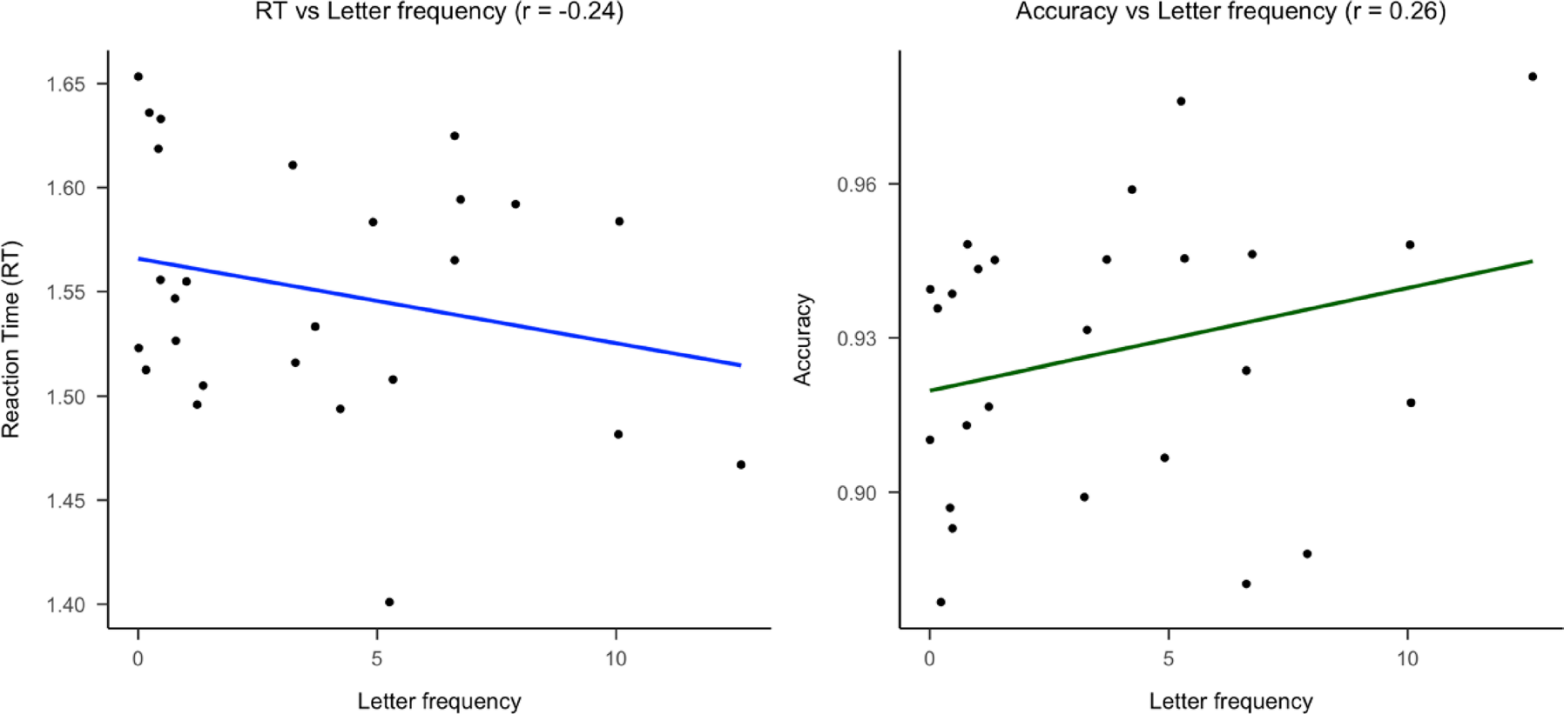
Correlations between letter frequency and performance measures for same-letter trials

Following a reviewer’s suggestion, we examined the possible influence of letter frequency on Braille letter discriminability. Because frequency is defined at the single-letter level, the analysis was restricted to same-letter trials. For these trials, in Fig. 10, we found a small negative correlation between letter frequency (computed from the Spanish database B-Pal, Davis & Perea, 2005) and reaction times and a positive correlation with accuracy, indicating that high-frequency letters were processed faster and more accurately, consistent with previous findings in visual reading (New & Grainger, 2011). Although such an effect could, in principle, extend to different-letter trials, if letter frequency had substantially influenced perceptual similarity, high-frequency letters would be expected to cluster together in the similarity analyses. However, the clusters were heterogeneous in terms of letter frequency, suggesting that frequency exerted only a minimal influence on the overall structure of braille letter similarity.

Overall, these findings suggest that tactile letter recognition is influenced not only by confusability (as revealed by “different” trials), but also by how efficiently a letter’s pattern can be processed in isolation. Thus, “same trials” provide an independent and theoretically important measure of perceptual fluency in braille reading.

## Discussion

The primary aim of the present study was to create a perceptual similarity matrix of Braille letters based on behavioral data, to serve as a benchmark resource for research on Braille reading and letter recognition. This resource is useful at multiple levels: It allows for precise control of stimulus similarity in experimental design, facilitates model development, and supports applied efforts in braille education and technology.

To achieve this goal, we conducted a same-different discrimination task using all possible pairs of braille letters, which is a method extensively validated in visual letter perception research for quantifying perceptual similarity (e.g., Courrieu et al., 2004; Mueller & Weidemann, 2012; Wiley et al., 2016). Based on participants’ responses, we constructed dissimilarity matrices from both accuracy and response time data, followed by hierarchical clustering analyses. This approach yielded a fine-grained view of the perceptual structure of braille letters, revealing consistent groupings across measures.

The results revealed that braille letters were consistently organized into four perceptual clusters. In the first cluster (*n *, *z *, *o *, and *y*), letters shared raised dots in every row and column except for a consistent gap at dot 2, while dot 5 was always raised, distinguishing them from other groups. The second cluster (*s **, t **, r **, w **, p *, and *q*) was defined by having raised dots across both columns and all rows, with raised dot 2 serving as a key feature separating them from other clusters. The third cluster (*m *, *x *, *k *, and *u *) was characterized by a distinctive gap in the middle row (dots 2 and 5). The fourth cluster (*i **, j **, d **, e **, a **, c **, b **, f **, g *, and *h *) included the ten base letters of the braille alphabet which were marked by consistent gaps in dots 3 and 6.

It is important to address the differences between visual and tactile letter similarity when interpreting these results. Previous research on visual letter similarity has shown that confusability patterns are largely driven by shared geometric features such as line orientation, curvature, or terminations, with letters like E–F–P or O–Q–C–G forming clusters based on visual overlap (Mueller & Weidemann, 2012; Simpson et al., 2012; see Wiley et al., 2016, for cross-script differences in Arabic). In braille, however, the similarity structure is shaped by tactile discriminability: Visually confusable letters are easily distinguished by touch, whereas confusion occurs between letters that belong to the tactile clusters identified in our results—such as d–f–h, u–v–x, or m–n–p. Thus, the patterns found here are not directly comparable to those reported in visual studies, indicating that braille letter similarity reflects modality-specific tactile constraints rather than visual geometry. Notably, even under speeded presentation with controlled timing (Table 1), we found reliable clusters in both accuracy- and RT-based analyses, indicating systematic tactile confusability among specific letters. This suggests that braille relies on modality-specific tactile features (dot-position patterns) that are rapidly integrated, but that these features still feed into an abstract orthographic code that may be shared with print (see also Reich et al., 2011 for fMRI evidence).

In addition, for fluent braille readers, there might be phonological activations that are automatic and might modulate the perceptual similarity. We believe that an analysis of these potential effects, while interesting, would require highly reliable phonological similarity norms, as the contribution of phonological similarity is likely to be small. In one of the few phonological similarity studies with European Spanish participants (not blind), García Lecumberri et al. (2013) reported a rather sparce pattern of misidentifications and showed that the most common confusions are between o and e, and e and o, which are not in the same cluster in our data.

The observed clusters appear to emerge in systematic ways that suggest underlying patterns or regularities; however, neither Millar’s emphasis on discrete dot positions and density nor Nolan and Kederis’s focus on holistic shape perception fully captures the perceptual organization found here. For instance, the consistent role of raised or absent dot in position 2 in the first group is in line with Millar’s (1977, 1985) suggestion that the location of individual dots exerts a strong influence on discriminability. Moreover, the observed groupings may also reflect more holistic or Gestalt-like shape processing. For example, if the absence of dots 2 and 5 in the third group is not perceived as two separate missing points but rather as a single continuous gap in the middle of the letter’s shape, this would support the argument by Nolan and Kederis (1969) that braille characters can be recognized as unified configurations instead of merely collections of individual features. This interpretation suggests that readers may rely on holistic spatial impressions, such as perceiving a “hole” or break in the overall form.

Same-trial data confirmed that letters with higher average dot informativeness were processed more quickly and accurately, complementing the confusability patterns observed in “different” trials and suggesting that informativeness facilitates rapid encoding, not just discrimination. These findings highlight the role of intrinsic feature structure in tactile fluency and complement the confusability data from “different” trials.

At the same time, the clustering patterns are not sufficient to determine whether recognition is driven mainly by local dot features, by global shape, or by an interaction of both. Most clusters differ by more than a single dot, implying that broader spatial arrangements contribute to discrimination alongside specific dot cues. Overall, the results point to a combined perceptual strategy: Individual dots remain informative but are integrated into higher-level structural representations. Another factor that may merit future investigation is the role of seriality in tactile processing. Certain dot positions (e.g., 4 and 6) might be less perceptually informative because they typically appear in conjunction with more salient positions (e.g., 1 and 3). Although we did not explicitly test this hypothesis, it suggests that the informativeness of individual dots may depend on their positional context within the braille cell given the way braille is read as shown in Fig. 5.

Although the present study was not designed to formally adjudicate among computational models of braille letter identification, the empirical clusters and dissimilarity matrices provide a grounded basis for future studies that can manipulate dot configurations more systematically and test how configuration-based and global-shape factors predict letter recognition. Furthermore, the matrices may also serve as input to process models, such as neural networks or diffusion models, that link behavioral data to mechanisms of tactile letter recognition. Critically, our approach could also be extended to contracted forms in braille (e.g., Grade 2 English, where high-frequency words like and ⠯ or for ⠿, graphemes like *th* ⠹, and affixes like -*er* ⠻ or -*ed* ⠫ can be represented by single cells), as well as structurally different braille systems (e.g., Japanese which is mostly syllabic based, or Korean which is an adaptation for Hangul script).

Beyond theoretical modeling, our letter similarity matrix has practical relevance for braille literacy instruction. Because some letters are more easily distinguished than others, one hypothesis is that early teaching might avoid introducing multiple highly confusable letters from the same cluster at the same moment. Instead, initial practice could contrast letters from different clusters to maximize perceptual distinctiveness. This suggestion is broadly consistent with work in category learning showing that interleaved exposure to contrasting exemplars can sharpen discrimination among confusable categories (Carvalho & Goldstone, 2015, 2017), although blocked presentation of similar items can sometimes help learners extract category internal commonalities. We therefore frame this proposal not as a fixed instructional prescription, but as an empirically testable prediction generated by the structure of the similarity matrices. For novice and late blind learners, who face particular challenges due to reduced tactile sensitivity or reliance on prior visual strategies, the matrix also highlights which letters hinge on the most informative dots and thus may require targeted practice.

## Conclusion

We have presented a perceptual similarity matrix for braille letters based on empirical data from fluent blind readers. This matrix captures consistent patterns in tactile confusability and reveals the influence of both local dot features and global configurations. It provides a valuable resource at multiple levels, supporting future modeling efforts, enabling better control of letter similarity in experiments, and informing braille literacy instruction. In addition, most visually impaired people in the world “outlive their eyes” and thus develop the need to learn braille at a stage of life when tactile sensitivity is greatly reduced. Future studies examining how reduced tactile sensitivity impacts letter confusability would be an important next step in this line of research.

### Public significance

Teachers, rehabilitation specialists, and device makers often ask a simple question with a hard answer: Which braille letters are easiest (or hardest) to tell apart by touch? There has been little empirical evidence to answer that question. In this study, we measured how similar every pair of letters in braille feels to fluent blind readers. Using a controlled setup with a refreshable braille display, readers decided whether two letters were the same or different while we recorded accuracy and response times for all possible pairs. From these data, we created a perceptual similarity matrix and identified clusters of letters that tend to be confused (or kept apart). We also compared these behavioral patterns with information-theoretic properties of dot positions to see which features help people most in practice. We found that tactile similarity is structured: Some letters form clear clusters, and specific dots carry more diagnostic value than others. This pattern goes beyond simple dot overlap, capturing how readers integrate local features into global structures. These findings can help researchers and educators about sequence letters in a way that reduces early confusions; for instance, early practice might contrast letters from different clusters rather than introducing multiple highly confusable letters at once. The perceptual similarity matrix also supports experimental control, and it provides a foundation for models of tactile letter and word recognition.

## Data Availability

The stimuli, data, scripts, and outputs are available at https://osf.io/98ek7/?view_only=89a6a4203f6b4450ae93e4d0033635e3.

## Appendix A. Correlation between accuracy- and RT-based dissimilarity matrices

.

## Footnotes

1 We use the lowercase term braille in accordance with the guidelines of the Braille Authority of North America (2006), which treats it as a common noun when referring to the writing system. When we refer to a braille character, we add the Latin letter in italics like this: *a *, *b *, *c *, etc. for reference.

2 Although we believe that studying Braille is important on its own right, without the need to triangulate through visual word recognition explanations (see Englebretson et al., 2023 for a compelling manifesto), in this article we refer to the visual word recognition literature when they inform our methods and/or the theoretical positions under examination.

## Code availability

The stimuli, data, scripts, and outputs are available at https://osf.io/98ek7/?view_only=89a6a4203f6b4450ae93e4d0033635e3.

## Open practices

The code for the simulations and the analyses is available at https://osf.io/98ek7/?view_only=89a6a4203f6b4450ae93e4d0033635e3.
